# Taxonomic notes on *Scutellaria taipeiensis* (Lamiaceae) from morphological and molecular data

**DOI:** 10.3897/phytokeys.140.48578

**Published:** 2020-02-24

**Authors:** Chien-Ti Chao, Bing-Hong Huang, Jui-Tse Chang, Pei-Chun Liao

**Affiliations:** 1 School of Life Science, National Taiwan Normal University, No. 88, Tingchou Rd. 4 section, Wenshan District, Taipei City 116, Taiwan National Taiwan Normal University Taipei City Taiwan

**Keywords:** Lamiaceae, Scutellarioideae, plant taxonomy, Taiwan

## Abstract

The genus *Scutellaria* comprises eight species distributed from 50 to 2000 m in Taiwan. Amongst them, *S. barbata* and *S. taipeiensis* are very similar on the basis of morphological and plastid DNA sequence information. Therefore, a comprehensive study of the taxonomic status of *S. taipeiensis* is necessary. We reviewed the herbarium sheets, related literature and protologues and compared morphologies of these two species, as well as their phylogenetic relationships. All evidence, including the diagnostic characters between *S. taipeiensis* and *S. barbata*, suggest that they belonged to a single species rather than two. As a result, *S. taipeiensis* is treated as a synonym of *S. barbata*.

## Introduction

The genus *Scutellaria* L. is composed of approximately 360 species worldwide (Paton 1990; Li and Hedge 1994; Harley et al. 2004). This genus is characterised by being non-aromatic, having simple leaves with entire to pinnatifid margins, a terminal or axillary raceme-like thrysoid inflorescence with single-flowered cymes, a two-lobed calyx with a scutellum on the upper lobe and a two-lobed corolla with an often saccate or spurred base, anterior anthers dimidiate due to aborted development of upper thecae and the ovary being borne on a peg-like gynophore (Paton 1990; Li and Hedge 1994).

The Taiwanese *Scutellaria* were revised in the 1990s, based on morphology and palynology and five species were recognised (Hsieh and Huang 1995). Later, a new species, *S. austrotaiwanensis* T. H. Hsieh & T. C. Huang was described (Hsieh and Huang 1997), resulting in a total of six species recorded in the second edition of the Flora of Taiwan (Huang et al. 1998). Two new species, *S. taipeiensis* T. C. Huang, A. Hsiao & M. J. Wu and *S. hsiehii* T. H. Hsieh, were described subsequently (Huang et al. 2003; Hsieh 2013). A genetic study of *S. barbata* D. Don and *S. taipeiensis* was conducted by Hsiung et al. (2017) and the data showed no remarkable divergence between these two species. These results attracted our attention to verify their findings. Therefore, we revised the taxonomic status of *S. taipeiensis* after re-evaluating morphological and plastid DNA sequence evidence in this study.

## Materials and methods

### Morphological comparison

Study materials were obtained from herbarium sheets of the HAST, TAI and TAIF herbaria and from living plants (herbarium acronyms follow Index Herbariorum (Thiers 2019, continuously updated). Type specimens of *S. taipeiensis*, deposited in the herbarium of the National Taiwan University (TAI), were also examined. Voucher specimens were deposited in the herbarium of the Taiwan Forestry Research Institute (TAIF). We examined leaf, floral and fruit morphology from dried and living materials. For living materials, we observed four populations of *S. barbata* and two of *S. taipeiensis*, including the type locality. *Scutellaria barbata* is widespread in Taiwan. Hence, herbarium sheets complement the fresh material gathered so that the variation, present in Taiwan, was represented in the study. For the population of *S. taipeiensis*, only few populations, including the type, have been recorded. All of these populations were located in Taipei City. Thus, our observation covered all populations in Taiwan. Observation of the nutlet sculpture of Hsiung et al. (2017) was applied here as a reference. The identification of *S. barbata*, *S. taipeiensis* and other Taiwanese species was according to the protologues of Huang et al. (2003) and other related literature (Hsieh and Huang 1995; Huang et al. 1998).

### Molecular analysis

In order to revise the taxonomic state of *S. taipeiensis*, phylogenetic trees were reconstructed. The species, selected for analysis, were from Chiang et al. (2012) and *Holmskioldia sanguinea* was applied as outgroup, since it was closely related to *Scutellaria* (Bendiksby et al. 2011). Two nuclear (CAD, CHS) and three chloroplast DNA fragments (*mat*K, *ndhF-rpl32 and rpl32-trnL*) were used by Chiang et al. (2012), amongst them, *ndhF-rpl32* and *rpl32-trnL* being also applied in the study of Hsiung et al. (2017). Two chloroplast regions (*ndhF-rpl32* and *rpl32-trnL*) were applied in the phylogenetic analysis of this study. In addition to the sequences from Chiang et al. (2012) and Hsiung et al. (2017), we sequenced the chloroplast DNA fragments of *ndh*F-*rpl*32 spacer from *S. galericulata*, *S. incana* and *H. sanguinea* and *rpl*32-*trn*L spacer from *S. galericulata*, *S. incana*, *S. taiwanensis* and *H. sanguinea*. These newly generated sequences were amplified following the procedure of Hsiung et al. (2017). All sequences, applied in this study, are listed in Table 1. These sequences were used for phylogeny reconstruction by Bayesian Inference (BI), Maximum Likelihood (ML) and Neighbour-Joining (NJ) approaches. The variable sites, parsimony-informative sites and substitution model were checked and selected by MEGA 7 (Kumar et al. 2016). The optimal model with the highest BIC and AIC values was selected for BI and ML analyses (Kumar et al. 2016) (Table 2). The BI reconstruction was conducted using Mr. Bayes 3.2.6 (Ronquist et al. 2012). Two independent runs were conducted with 10,000,000 generations, sampled every 1000 generations and a 10% dataset was discarded as burn-in. ML analysis was performed by PhyML 3.1 (Guindon et al. 2010). The substitution model of the two loci was the same as for BI analysis and the gamma distribution parameter was fixed at 1.52 and 0.35, respectively, according to the results of model selection. The tree topology search operation was set as the best of NNI and SPR (Guindon et al. 2010). The approximate likelihood ratio test non-parametric branch support was based on a Shimodaira-Hasegawa-like procedure (Guindon et al. 2010). NJ analysis was conducted using MEGA 7, with 1000 bootstrap resamplings. All of the phylogenetic trees were summarised and output by FigTree 1.4.4 (Rambaut 2012).

**Table 1.** Sequences and accession number of sequences applied in this study. Sequences generated for this study are marked *. Other sequences were sourced from Genbank.

Scientific name	*ndh*F-*rpl*32	*rpl*32-*trn*L
*Scutellaria barbata*	KY458956.1	KY458962.1
	KY458957.1	KY458963.1
	KY458958.1	KY458965.1
	KY458959.1	KY458966.1
*S. alpina*	JX981401.1	JX981439.1
*S. baicalensis*	JX981400.1	JX981443.1
*S. altissima*	JX981404.1	JX981440.1
*S. zhongdianensis*	JX981405.1	JX981441.1
*S. diffusa*	JX981406.1	JX981442.1
*S. galericulata*	MN720754*	MN720750*
*S. incana*	MN883839*	MN883840*
*S. lateriflora*	JX981403.1	JX981444.1
*S. indica*	JX981422.1	JX981387.1
	JX981423.1	JX981388.1
*S. austrotaiwanensis*	JX981421.1	JX981386.1
	JX981429.1	JX981394.1
	JX981430.1	JX981393.1
	JX981431.1	
	JX981432.1	
*S. tashiroi*	JX981433.1	
*S. playfairii*	JX981424.1	JX981389.1
	JX981425.1	JX981390.1
	JX981426.1	JX981391.1
		JX981392.1
*S. salviifolia*	JX981402.1	JX981438.1
	JX981427.1	MN720752*
*S. taiwanensis*	JX981428.1	MN720753*
	KY458960.1	KY458964.1
*S. taipeiensis*	KY458961.1	KY458967.1
*Holmskioldia sanguinea*	MN720755*	MN720751*

**Table 2.** Summary of the sequence information of two plastid region applied in phylogenetic analysis.

	*ndh*F-*rpl*32	*rpl*32-*trn*L
Aligned length (bp)	536	546
No. of variable characters	195	120
No. of parsimony-informative characters	117	58
Substitution model	HKY+G	HKY+G

## Results

### Diagnostic characters of *S. barbata* and *S. taipeiensis*

Leaves

**Figure 1.**
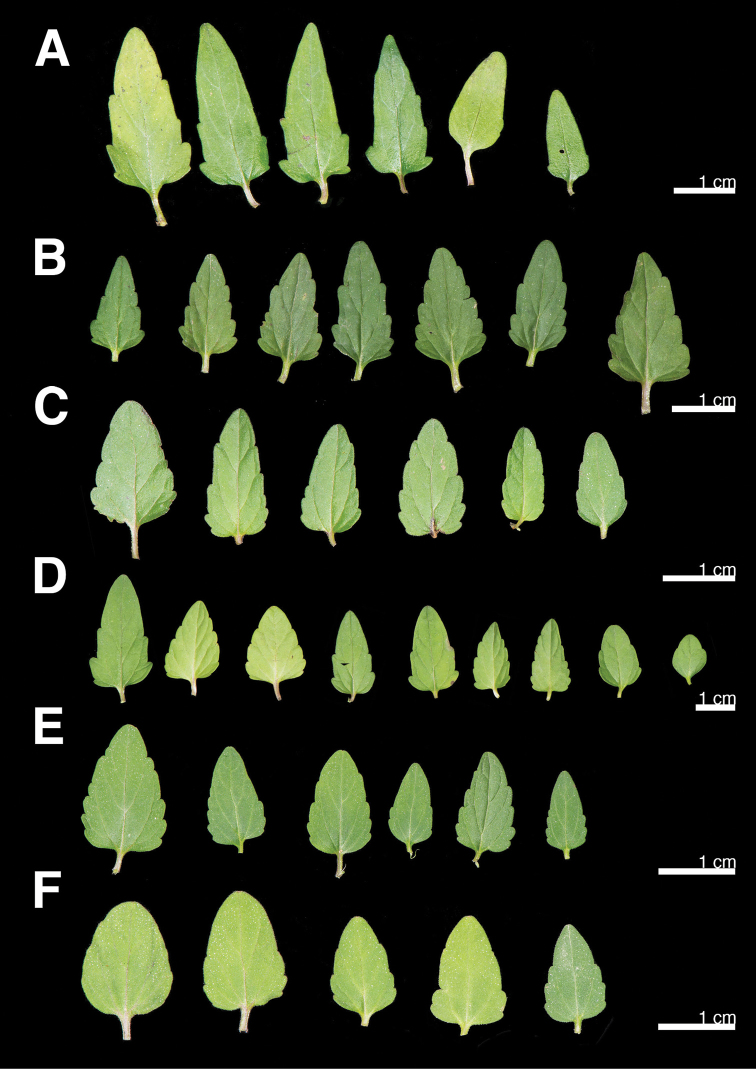
Leaf variation of *S. barbata* (**A–D**) and *S. taipeiensis* (**E, F**). **A** New Taipei City, Gueishan rd. (Chao 4768) **B** Taipei City, Hsichou street (Chao 4762) **C** Ilan County, Ilan City (Chao 4787) **D** Ilan County, Sanhsing Township (Chao 4789) **E** Taipei City, campus of NCCU (Chao 4837) **F** Taipei City, Maukong (type locality, Chao 4838).

Leaf morphology had been regarded as a diagnostic character for distinguishing *S. barbata* from *S. taipeiensis* (Huang et al. 2003). Leaf shape of *S. barbata* varies from suborbicular to narrowly lanceolate (Fig. 1A–D); in contrast, the leaf shape of *S. taipeiensis* varies from ovate to broadly ovate (Fig. 1E, F). The leaves of both species had sparse pubescence on the abaxial surface. The leaves of *S. barbata* are 1.1−2.8 cm long and 0.9−1.4 cm wide, the length-width-ratio from 1.1 to 2.0, while *S. taipeiensis* leaves are 1.0−1.7 cm long and 0.5−1.1 cm wide, the length-width-ratio from 1.5 to 2.0. The shapes and sizes of the leaves overlapped between the two species and thus were difficult for use as a diagnostic character to distinguish species.

Inflorescence and flowers

**Figure 2.**
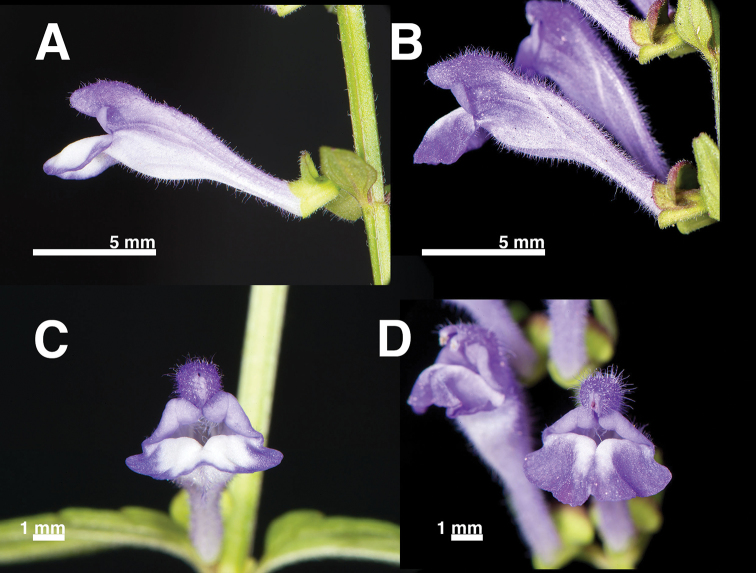
Flower morphology of *S. barbata* (**A, C**)(Chao 4762) and *S. taipeiensis* (**B, D**)(Chao 4838). **A, B** lateral view **C, D** front view.

**Figure 3.**
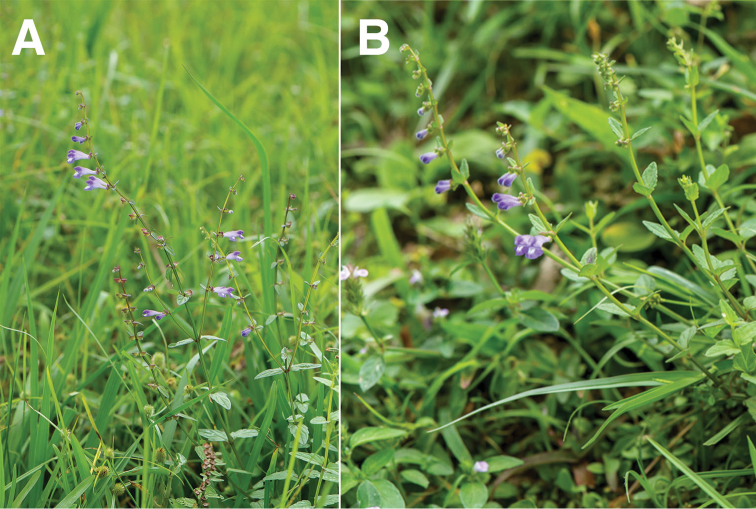
Inflorescence morphology of *S. barbata* (**A**) and *S. taipeiensis* (**B**).

The floral morphology of *S. barbata* (Fig. 2A, C) was very similar to that of *S. taipeiensis* (Fig. 2B, D). They both had terminal inflorescences and bilabiate flowers that were only slightly curved near base (Fig. 3), while other species of Taiwan have geniculate (e.g. *S. austrotaiwanensis*, *S. indica* etc.) or a strongly curved corolla (e.g. *S. tashiroi*). The corolla was bluish-purple, 0.8−1.3 cm long and pubescent on the outer surface.

Nutlets

According to the observations of Hsiung et al. (2017), the sculpture of *S. barbata* and *S. taipeiensis* are rounded concentric type and no other difference is found between them.

Molecular phylogeny

**Figure 4.**
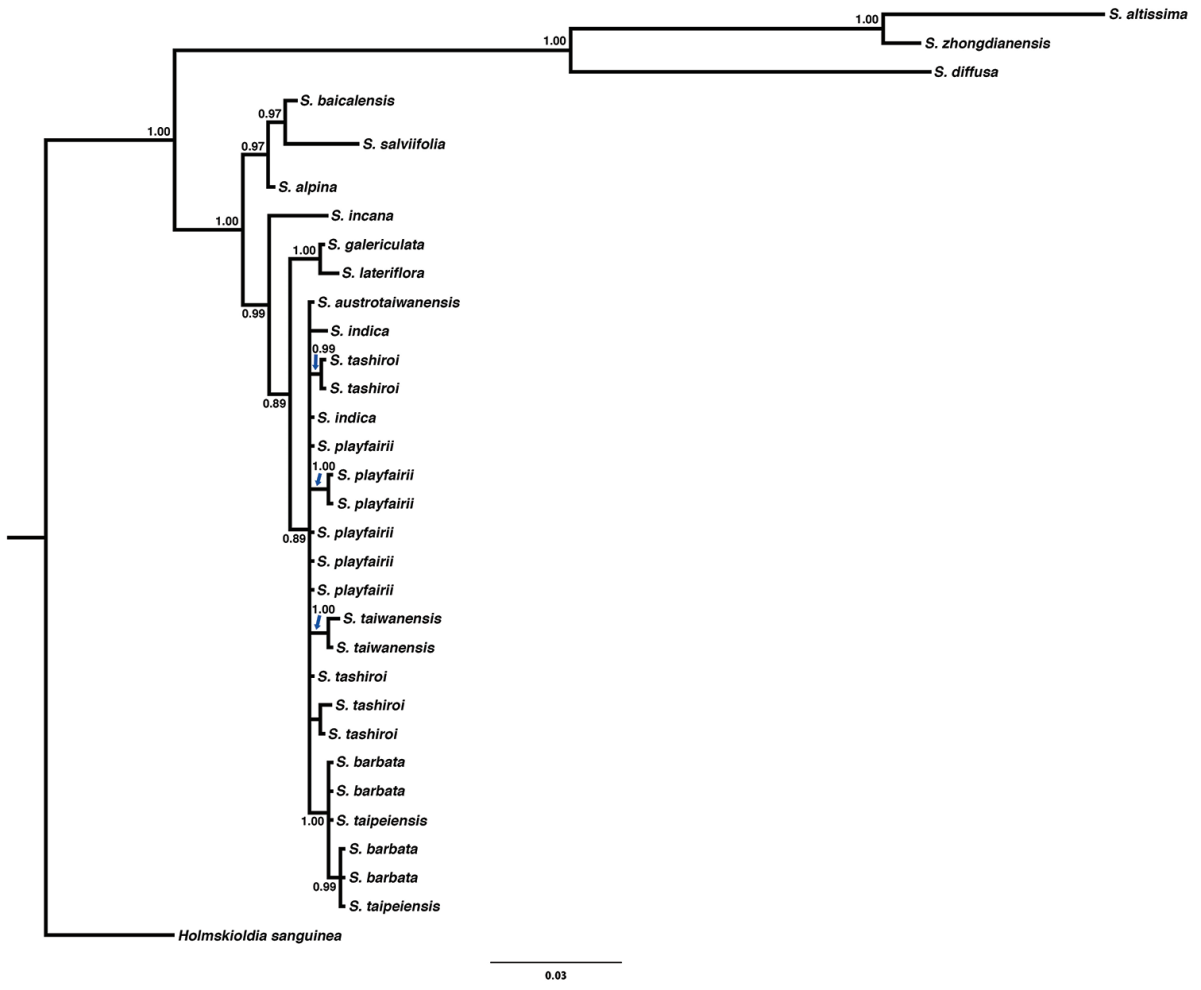
Phylogenetic tree reconstructed by Bayesian inference from chloroplast DNA sequence *ndh*F-*rpl*32. Only posterior probability > 0.85 was labelled on the branch. Scale bar represent substitutions.

**Figure 5.**
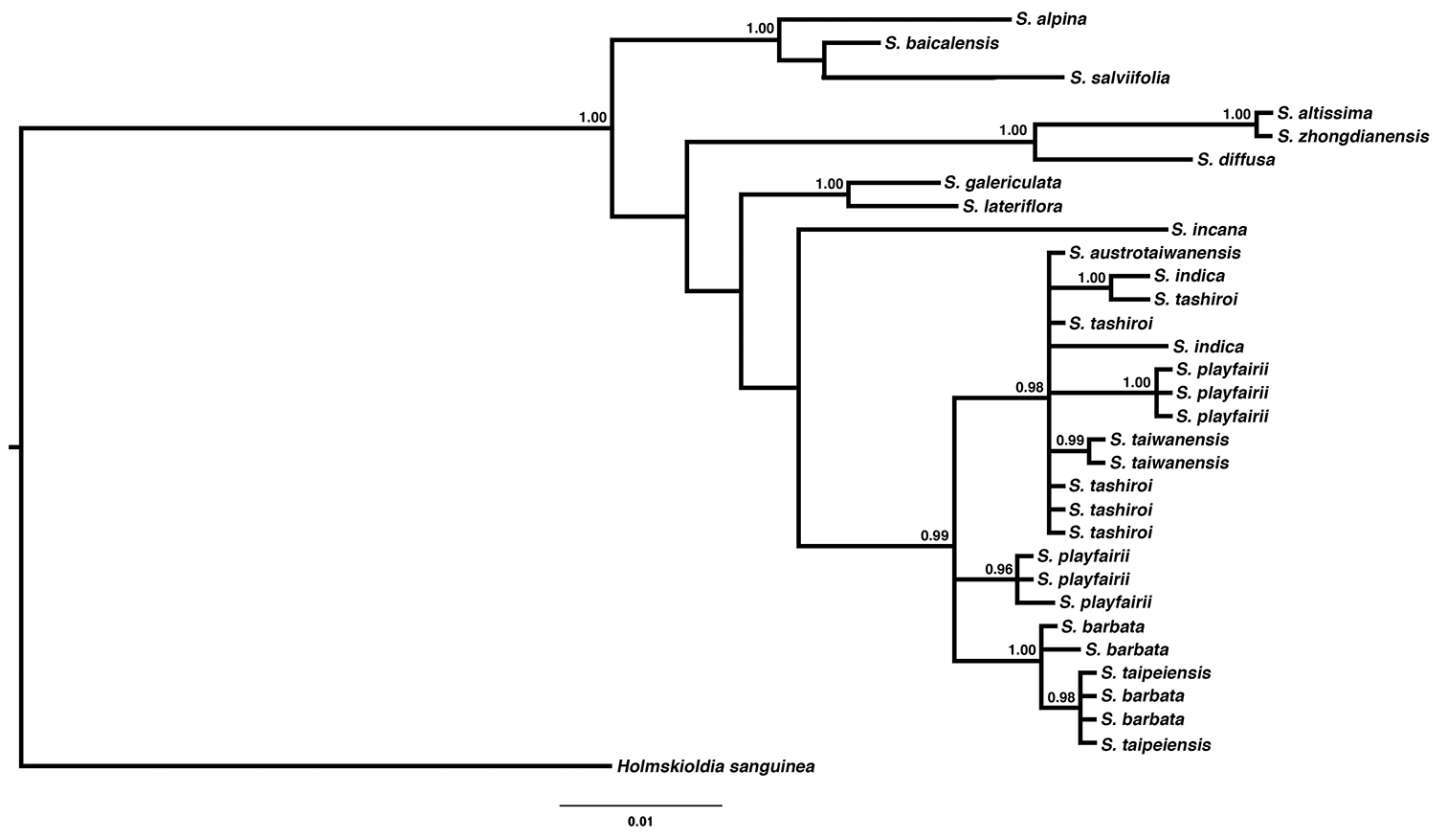
Phylogenetic tree reconstructed by Bayesian inference from chloroplast DNA sequence *rpl*32-*trn*L. Only posterior probability > 0.85 was labelled on the branch. Scale bar represent substitutions.

The best substitution model for both fragments, *ndh*F-*rpl*32 and *rpl*32-*trn*L, was HKY+G. The phylogenetic trees, reconstructed by ML, NJ and BI, revealed similar topologies with slight differences in the arrangement of non Taiwanese species (Figs 4, 5; Suppl. materials 1–4). In all analyses, all Taiwanese species formed a clade with moderate to high support (PP = 0.99, bootstrap = 0.65–0.91). Amongst them, *S. barbata* and *S. taipeiensis* formed a highly supported monophyletic group (PP = 1.0, bootstrap = 0.87–0.95), but neither *S. barbata* nor *S. taipeiensis* formed a single clade. Instead, *S. taipeiensis* was nested with *S. barbata* in the phylogenetic tree, i.e. neither species being monophyletic as currently delimited.

Distribution and habitat

*Scutellaria barbata* grows throughout low altitudes, ca. 50–500 m, in Taiwan, but lower in southern parts. *Scutellaria taipeiensis* is found in Taipei City and New Taipei City. The two species were growing in similar habitats, such as grassland, roadside, riverbank or forest margin and often with high humidity. Some references regarded *S. barbata* as an aquatic plant in a broad sense (Chen 1990) due to its wetland habitat. This could also be true for *S. taipeiensis*, according to the field investigation. No apparent differentiation was observed in the distribution and habitat types between these two species.

## Discussion

### The taxonomic status of *S. taipeiensis*

*Scutellaria taipeiensis* was first described by Huang et al. (2003), based on the morphology of the leaves and the nutlets. According to the original description, the leaves of *S. taipeiensis* were triangular-ovate to broadly ovate and less than twice as long as the width. To confirm the similarity of the leaves, we collected and compared leaf morphology of the two species amongst several populations. The results showed that the variation within *S. barbata* was larger than the difference between *S. barbata* and *S. taipeiensis*. Additionally, the length-width-ratio of leaves is the same in both species. Therefore, leaf morphology could not be regarded as a diagnostic character for these two species.

With regard to the nutlets, the coat had also been used to distinguish *S. barbata* from *S. taipeiensis*. The former had a radiating, umbrella-like shape, while the latter was a rounded concentric type (Huang et al. 2003). However, Hsiung et al. (2017) reviewed this character on a population level and found no remarkable difference between the two species. The mature nutlets appear to the rounded concentric type in both species, such state was stable amongst populations (Hsiung et al. 2017). The umbrella-like appendage was found in immature nutlets only, which meant that it was a transitional state during nutlet development and could not provide a valid taxonomic value.

We further looked at other characters to separate them, such as floral morphology and DNA sequence data. Different sequence data revealed some phylogenetic incongruence amongst lineages *S. alpina*, *S. altissima*, *S. bicalensis*, *S. diffusa*, *S. galericulata*, *S. salviifolia* and *S. zhongdianensis*. Such incongruence may be due to uneven sampling, but the relationship of these species was not a concern in this study. Therefore, we will not discuss the evolutionary relationship between this group of species here. All Taiwanese species formed a highly supported clade in all trees. *Scutellaria barbata* and *S. taipeiensis*, which had very similar inflorescences and floral morphology, are phylogenetically nested within a monophyletic clade. Based on this evidence, *S. taipeiensis* was treated as a synonym of *S. barbata*, rather than a distinct species or on an intraspecific level.

### Taxonomic treatment

According to the results and discussions, we established the following taxonomy:

#### Scutellaria barbata

Taxon classificationPlantaeLamialesLamiaceae

D. Don in Prodromus Florae Nepalensis 109–110. 1825.

124A9FC4-4194-5DD0-9347-0830267E8620

S. taipeiensis T. C. Huang, A. Hsiao & M. J. Wu in Taiwania 48:133. Type: Taiwan. Taipei City, growing on exposed rocks or soils adjacent rocks along sunny roadside between Maukong to Chihnan Temple T. C. Huang and A. Hsiao 18104 (Holotype: TAI!, Isotype: TAI!) syn. nov.

##### Distribution.

*Scutellaria barbata* is widely distributed in southern and eastern Asia (Li and Hedge 1994). In Taiwan, this species is found in low altitude from 50 to 500 m, in wet grasslands, riverside and margins of forest.

##### Specimens examined.

Specimens marked with an asterisk (*) denote material *S. taipeiensis* following.the concept of Huang et al. (2003) on the labels.

**Taiwan. Changhua County**: Lukang, at road mileage sign 35 km along Provincial Highway 17, 18 Apr 1999, K. F. Chung 1147 (HAST). **Hsinchu County**: Hengshan, Peiwuo, 245 m a.s.l., 28 Apr 1994, C. M. Wang 763 (HAST). **Hualien County**: *Fengping Township, a public cemetery, 0−50 m a.s.l., 1 May 2015, S. W. Chung 12187 (TAIF); Patu, 9 Jul 2008, M. J. Jung 3055 (TAIF); Fuli Township, 22 May 2012, S. H. Chen s.n. (TAIF); Juisuei Township, 28 Jan 1987, S. H. Chen s.n. (TAIF); Kaoliao, 12 Feb 1990, J. P. Lin 421 (TAIF). **Ilan County**: Shuanglien Pond, 250−300 m a.s.l., 10 Apr 2009, W. Y. Wang 153 (TAIF); Pitou Lake, 1 Apr 2012, S. Z. Tsai & Y. S. Liang TSY265 (TAIF); Tungshan Township, 6 Oct 1991, Y. H. Liou Liu9110A-027 (TAIF); Tali, 20 Apr 1962, C. C. Chuang 2171 (TAI); Kanchiaokeng, 8 Feb 2001, H. Y. Chen & K. L. Jien 1601 (TAI); Meihuahu, 50 m a.s.l., 23 May 2000, C. H. Lin 352 (HAST); Ilan City, Huanhe N. Rd., out of river bank, 23 Mar 2019, C. T. Chao 4787 (TAIF); Annong river flood diversion weir park, 23 Mar 2019, C. T. Chao 4789 (TAIF); Ilan, 100 m a.s.l., 22 Mar 1987, S. Y. Lu 21257 (TAIF); Panomakutao, 350 m a.s.l., 2 Apr 2005, W. F. Ho 1735 (TAIF); Shuanglien Pond, 250−300 m a.s.l., 10 Apr 2009, W. Y. Wang 153 (TAIF); Dongshan river, 50 m a.s.l., 13 Feb 2012, S. W. Chung 10589 (TAIF); Shan-shin, 7 Apr 1982, M. T. Kao 9656 (TAI). **Kaohsiung City**: Lienhuachih, 30 Aug 1991, L. Y. Tseng 509 (TAIF). **Keelung City**: *Tienwaitien Landfill Site, 150 m a.s.l., 25 Apr 2014, P. F. Lu 26638 (TAIF); Patu, 9 Jul 2008, M. J. Jung 3055 (TAIF). **Miaoli County**: Miaoli, 17 Apr 1970, T. C. Chuang 5277 (TAI). **Nantou County**: Chungyuan neighbourhood, 24 Jan 1988, S. M. Li 76 (TAIF). **New Taipei City**: *Mt. Erhke, 8 May 2011, M. J. Jung 5453 (TAIF); *Mt. Chungling, 400−600 m a.s.l., 10 Jan 2015, P. F. Lu 27688 (TAIF); Hsiaokotou-Kankou, 27 Dec 1968, C. C. Hsu 5213 (TAI); Wazihwei, 0−20 m a.s.l., 16 Apr 2004, S. C. Liu 1711 (TAIF); Sanchakang Village, 100 m, 4 Oct 2008, P. F. Lu 16991 (TAIF); Fujen Catholic University, 22 Dec a.s.l. 2002, C. L. Hu s.n. (TAIF); Santiaoling, 60 m a.s.l., 20 Apr 2012, S. W. Chung 10815 (TAIF); Menghu Rd., 26 Mar 2012, C. F. Chen 3306 (TAIF); Sanhsia, 5 Apr 1994, T. H. Hsieh 1194 (TAI); Hsiaokengkou, 50−100 m a.s.l., 19 May 2000, H. Y. Chen 1398 (TAIF); Shihting to Huangtitien, 1 Jun 2003, T. C., L. C. & R. P. Huang 18105 (TAI); Sshlioufennz, 300−370 m a.s.l., 4 Apr 1985, C. I Peng 7551 (HAST); Hsinshang-Menghu, 350−400 m a.s.l., 16 May 1993, C. C. Wang 1363 (HAST); Hsichou Street, 26 Feb 2019, C. T. Chao 4762 (TAIF); Gueishan rd., 15 Mar 2019, C. T. Chao 4768 (TAIF); Hsiunghustien to Peihsinchuang, 100 m a.s.l., 23 Mar 2001, S. M. Kuo 216 (TAIF); Shuangshi, 100 m a.s.l., 12 Jul 2003, P. F. Lu 5134, 6255 (TAIF); Yunhsien garden, 700−800 m a.s.l., 29 Mar 2000, Y. P. Cheng 2911 (TAIF); Gungliau, Waiwenshiouk, 50 m a.s.l., 9 Apr 2000, H. M. Chang 3110 (TAIF); HuangTiTien, 150−250 m a.s.l., 16 Apr 2011, P. F. Lu 21794 (HAST); Ta-li, 20 Apr 1962, C. C. Chuang 2171 (HAST); Chuwei, 26 Feb 1989, T. Y. Yang & C. C. Wang 4474 (TAI); Shihting, 20 Apr 1991, M. J. Wu 1303 (TAI); Yinhoton, 28 Aug 1970, M. T. Kao 7621 (TAI); Pinlin, 1 Apr 1977, C. M. Kuo 8119 (TAI). **Taichung City**: Pingting, 18 Jul 1968, C. C. Hsu 9128 (TAI); Fengyuan, along a steep trail between Panchang and Fengyuan Golf Club, 250−400 m a.s.l., 20 Dec 1985, C. I Peng 12165 (HAST); Ta-chia, 28 Apr 1982, M. T. Kao 9677 (TAI). **Taipei City**: *Huajiang Wild Duck Nature Park, 12 Apr 2010, M. J. Jung 4918; *same loc., 20 Apr 2010, M. J. Jung 4933 (TAIF); NTU farm, 14 Dec 1960, M. T. Kao 7633 (TAI); Shuiyuanti, H. Simizu 210 (TAI); Mucha, 25 May 1975, C. I Peng 1464 (TAI); *Maokun, 200 m a.s.l., 13 Apr 2003, T. C. Huang 18103 (TAI); at Tachia Riverfront Park, 15 m a.s.l., 21 Mar 2007, C. I Huang 3094 (HAST); Taipei, 10 Jul. 1908, Y. Simada s.n. (TAIF); same loc., Dec. 1909, Y. Kawakami & S. Sasaki s.n. (TAIF); Neihu, 16 Apr 1974, C. M. Kuo 4820 (TAI); Campus of NCCU, 13 May 2019, C. T. Chao 4837 (TAIF); Section 3 of Chinan Rd., 13 May 2019, C. T. Chao 4838 (TAIF). **Taitung County**: Provincial Rd. No. 11, 29 Mar 2003, Y. C. Liu s.n. (TAIF). **Taoyuan City**: Jeiyuan neighbourhood, 17 Dec 2005, I W. Deng s.n. (TAIF); Jenmei, 1 May 1975, C. M. Guo 6119 (TAI); Chungyuan Univ., 17 Mar 1976, C. M. Kuo 6613 (TAI);

## Supplementary Material

XML Treatment for Scutellaria barbata

## Supplementary materials

Supplementary material 1 Phylogenetic tree reconstructed by maximum likelihood analysis from chloroplast DNA sequence *ndh*F-*rpl*32. Only SH-like support value > 0.85 was labelled on the branches. Scale bar represent substitution Data type: molecular data. https://binary.pensoft.net/file/382406This dataset is made available under the Open Database License (http://opendatacommons.org/licenses/odbl/1.0/). The Open Database License (ODbL) is a license agreement intended to allow users to freely share, modify, and use this Dataset while maintaining this same freedom for others, provided that the original source and author(s) are credited.Chien-Ti Chao, Bing-Hong Huang, Jui-Tse Chang, Pei-Chun Liao

Supplementary material 2 Phylogenetic tree reconstructed by maximum likelihood analysis from chloroplast DNA sequence *rpl*32-*trn*L. Only SH-like support value > 0.85 was labelled on the branches. Scale bar represent substitution Data type: molecular data. https://binary.pensoft.net/file/382407This dataset is made available under the Open Database License (http://opendatacommons.org/licenses/odbl/1.0/). The Open Database License (ODbL) is a license agreement intended to allow users to freely share, modify, and use this Dataset while maintaining this same freedom for others, provided that the original source and author(s) are credited.Chien-Ti Chao, Bing-Hong Huang, Jui-Tse Chang, Pei-Chun Liao

Supplementary material 3 Phylogenetic tree reconstructed by neighbour-joining analysis from chloroplast DNA sequence *ndh*F-*rpl*32. Only bootstrap support value > 0.85 was labelled on the branches Data type: molecular data. https://binary.pensoft.net/file/382408This dataset is made available under the Open Database License (http://opendatacommons.org/licenses/odbl/1.0/). The Open Database License (ODbL) is a license agreement intended to allow users to freely share, modify, and use this Dataset while maintaining this same freedom for others, provided that the original source and author(s) are credited.Chien-Ti Chao, Bing-Hong Huang, Jui-Tse Chang, Pei-Chun Liao

Supplementary material 4 Phylogenetic tree reconstructed by neighbour-joining analysis from chloroplast DNA sequence *rpl*32-*trn*L. Only bootstrap support value > 0.85 was labelled on the branches Data type: molecular data. https://binary.pensoft.net/file/382409This dataset is made available under the Open Database License (http://opendatacommons.org/licenses/odbl/1.0/). The Open Database License (ODbL) is a license agreement intended to allow users to freely share, modify, and use this Dataset while maintaining this same freedom for others, provided that the original source and author(s) are credited.Chien-Ti Chao, Bing-Hong Huang, Jui-Tse Chang, Pei-Chun Liao
