# Supplementary figures and images for: Taxonomic notes on Scutellaria taipeiensis (Lamiaceae) from morphological and molecular data
Source: PhytoKeys. 2020 Feb 24;140:33–45. doi: 10.3897/phytokeys.140.48578 (PMC7052021; doi:10.3897/phytokeys.140.48578)

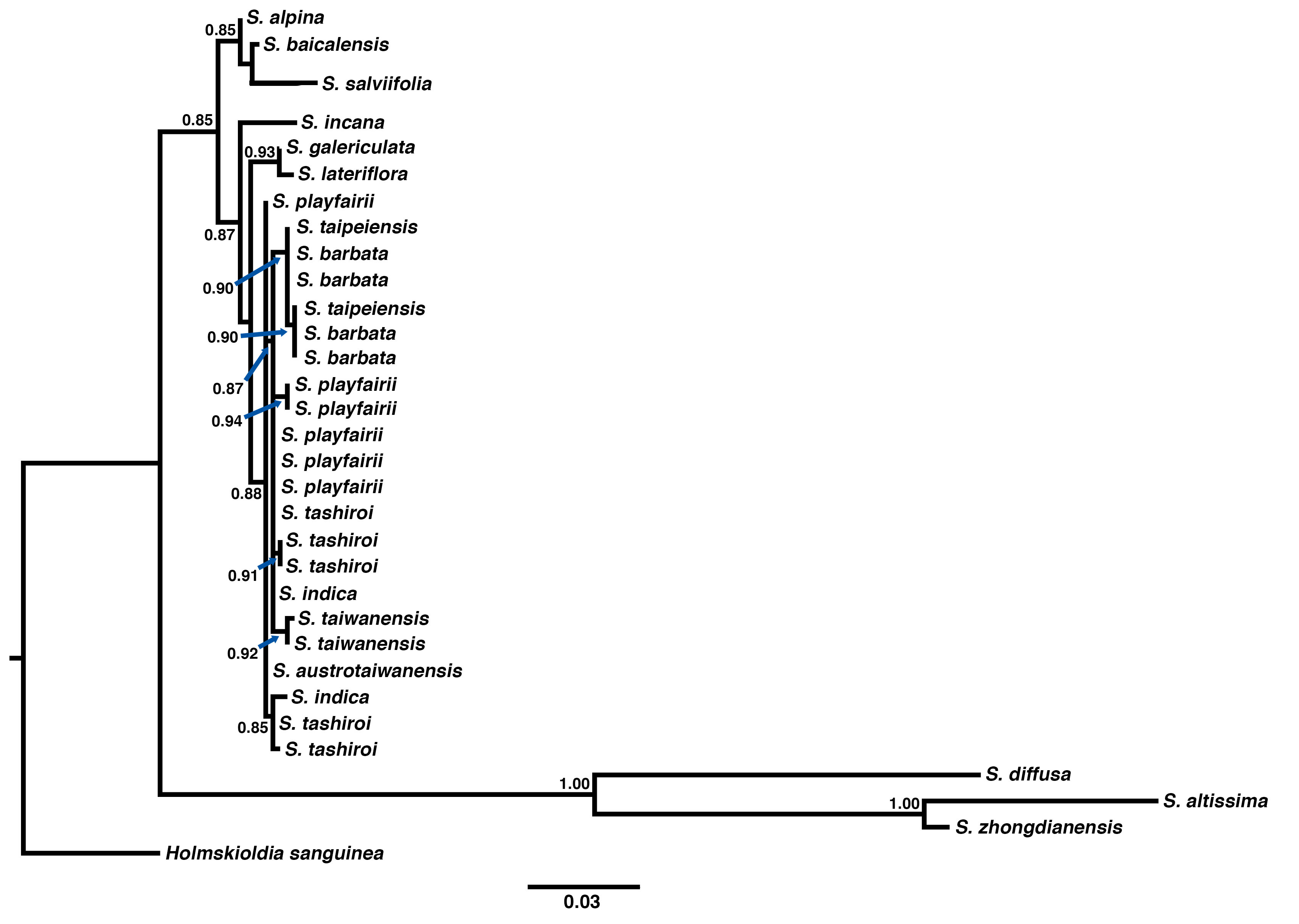

Supplement: Supplementary material 1 [file phytokeys-140-033-s001.jpg]

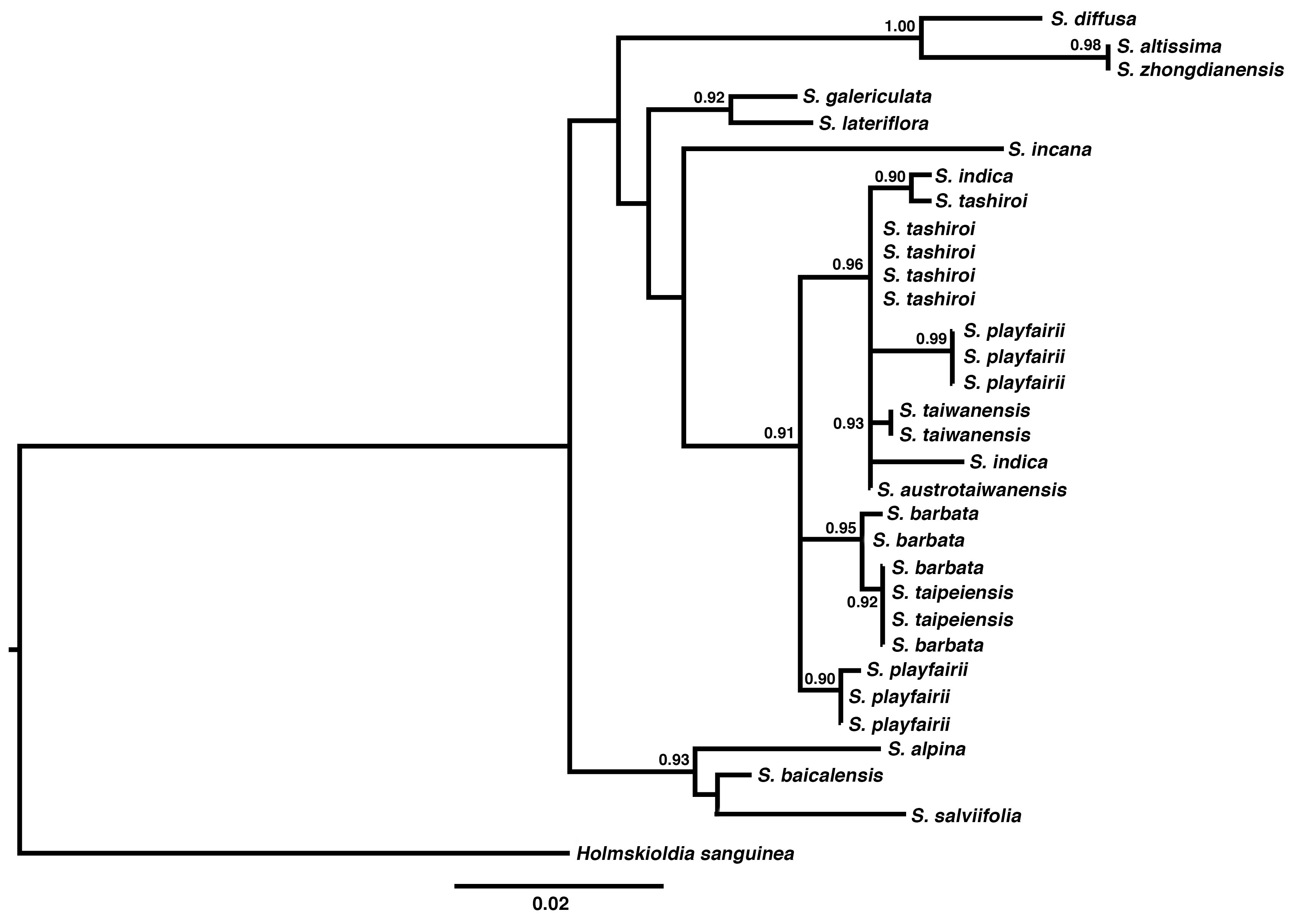

Supplement: Supplementary material 2 [file phytokeys-140-033-s002.jpg]

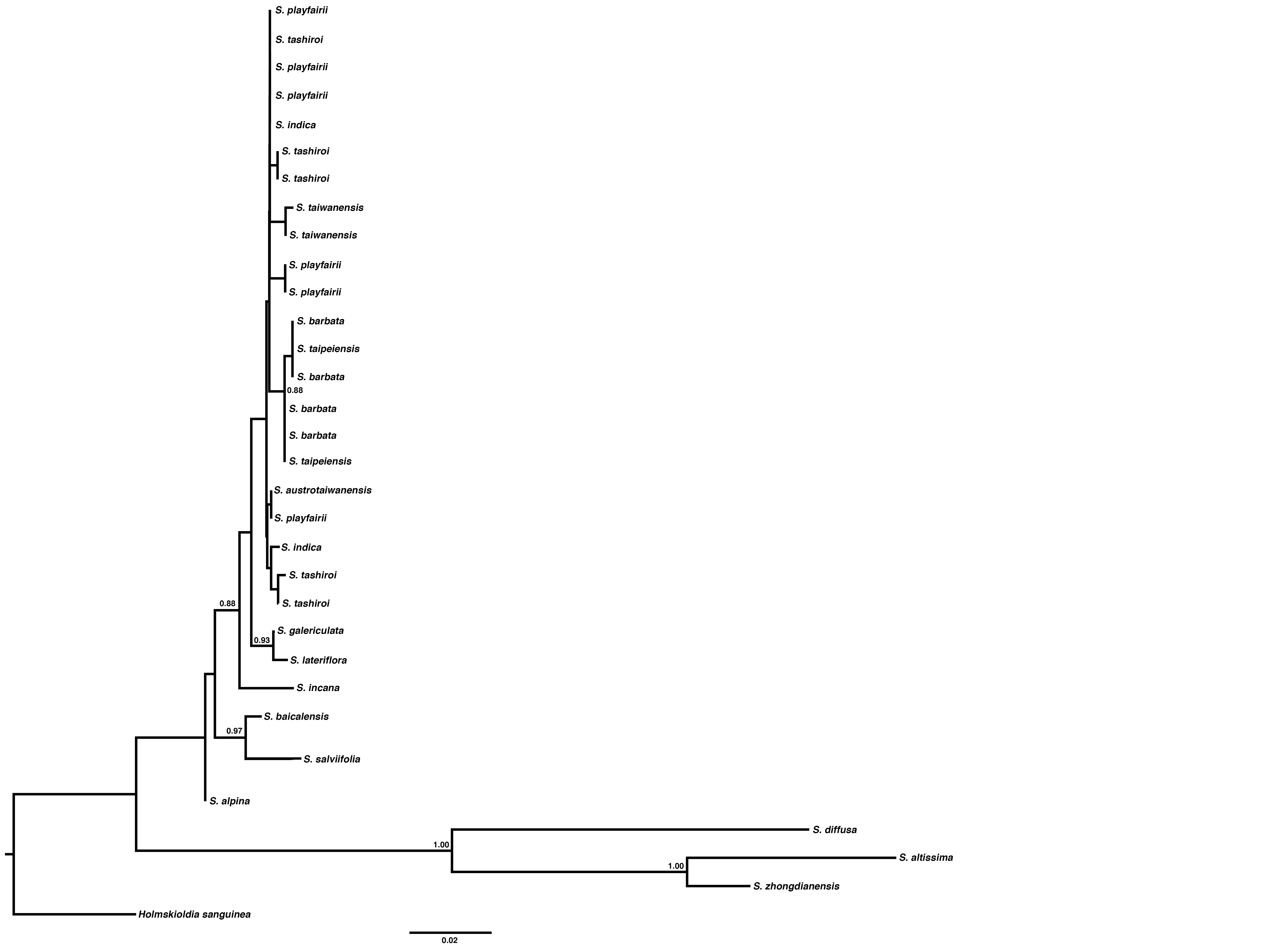

Supplement: Supplementary material 3 [file phytokeys-140-033-s003.jpg]

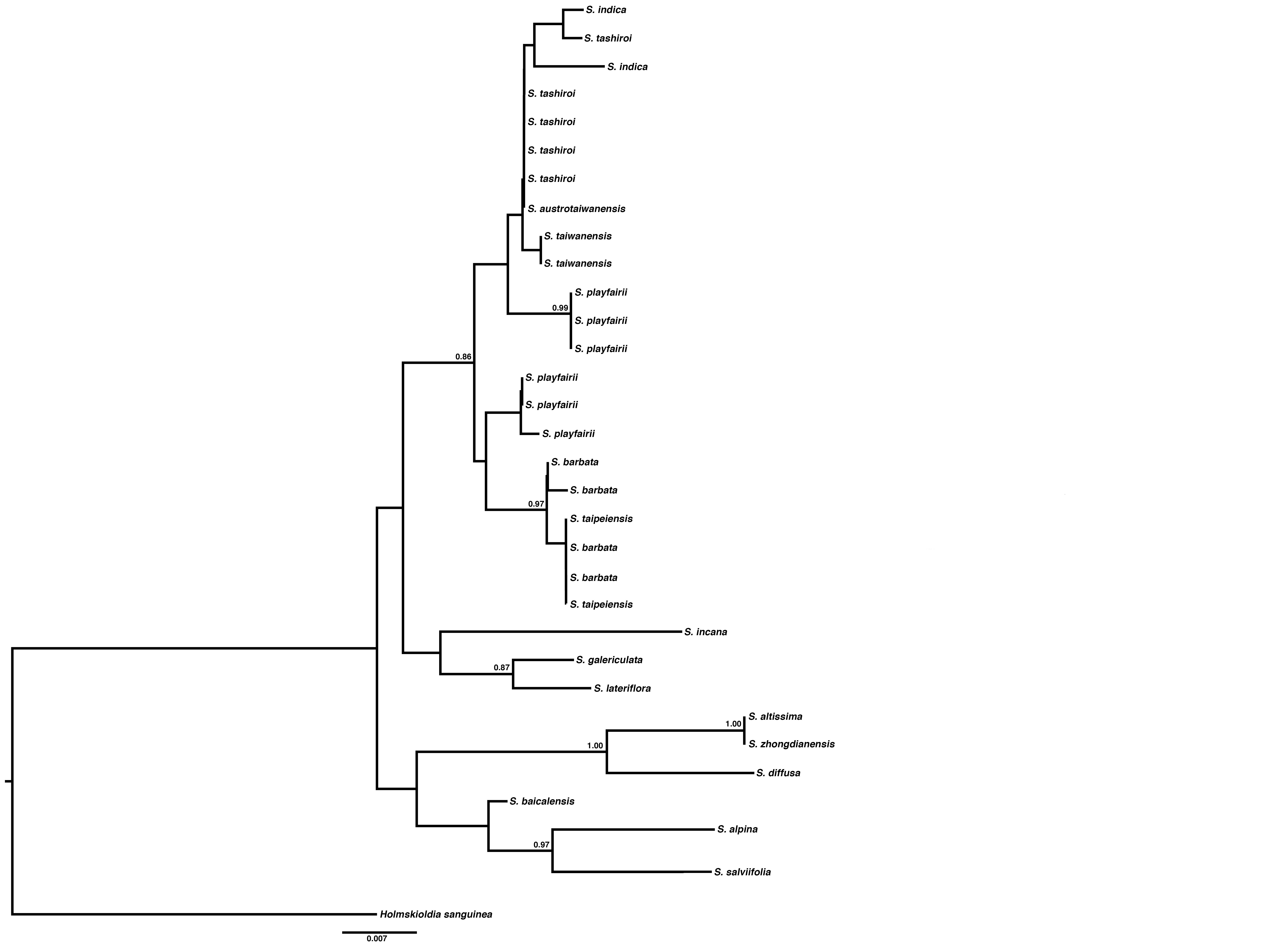

Supplement: Supplementary material 4 [file phytokeys-140-033-s004.jpg]
